# Effects of emixustat hydrochloride in patients with proliferative diabetic retinopathy: a randomized, placebo-controlled phase 2 study

**DOI:** 10.1007/s00417-020-04899-y

**Published:** 2020-08-27

**Authors:** Ryo Kubota, Chirag Jhaveri, John M. Koester, Jeffrey K. Gregory

**Affiliations:** 1 Acucela Inc., 818 Stewart St., Suite 1110, Seattle, WA 98101-1479 USA; 2 Retina Consultants of Austin, 3705 Medical Parkway, Suite 410, Austin, TX 78705 USA; 3 Department of Ophthalmology, Dell Medical School, University of Texas at Austin, 1601 Trinity St., Building B, Stop Z1200, Austin, TX 78712 USA

**Keywords:** Emixustat, Diabetic retinopathy, Cytokines, Diabetic macular edema, VEGF

## Abstract

**Purpose:**

To evaluate the effects of oral emixustat hydrochloride on pro-angiogenic and inflammatory cytokines in the aqueous humor, as well as other ophthalmic parameters, in subjects with proliferative diabetic retinopathy (PDR).

**Methods:**

Twenty-three patients with PDR, with or without diabetic macular edema (DME), were assigned to emixustat or placebo in daily oral doses ranging from 5 to 40 mg over a step-up titration period, for 84 days. The main outcome measures included levels of IL-1β, IL-6, IL-8, TGFβ-1, and VEGF in the aqueous humor.

**Results:**

Seven of 12 subjects (58%) who were randomized to emixustat and 11 of 12 subjects (92%) who were randomized to placebo completed the study. No statistically significant differences between treatment groups were observed for changes in any of the aqueous humor cytokines tested. However, median VEGF levels were slightly reduced in the emixustat but not the placebo group (− 70.0 pg/mL versus + 42.7 pg/mL, or − 11.8% versus + 6.7%). In a post hoc analysis of all subjects (with or without DME), statistically significant differences between treatment arms in mean changes from baseline in central subfield thickness (CST; emixustat − 11.9 μm, placebo + 36.2 μm; *P* = 0.076) and total macular volume (TMV; emixustat − 0.13 mm^3^, placebo + 0.23 mm^3^; *P* = 0.026) were observed, both favoring emixustat. Emixustat’s safety profile was consistent with prior studies (i.e., the adverse events of delayed dark adaptation and visual impairment were more common in subjects treated with emixustat).

**Conclusion:**

Although this pilot study did not demonstrate statistically significant differences in changes in aqueous humor cytokine levels between the emixustat and placebo groups, VEGF levels were slightly reduced in the emixustat but not in the placebo group. In addition, statistically significant differences favoring the emixustat group were observed in CST and TMV among all subjects. These data warrant further investigation of emixustat’s potential therapeutic effects in diabetic retinopathy.

**Trial registration:**

ClinicalTrials.gov identifier: NCT02753400 (April 2016)

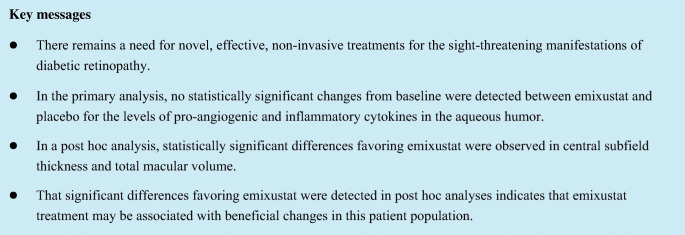



## Introduction

Diabetic retinopathy (DR), particularly in the forms of proliferative diabetic retinopathy (PDR) and diabetic macular edema (DME), is the most common cause of blindness in the US working population [1] and can reduce individuals’ independence, mobility, and quality of life [2]. Recently, anti-vascular endothelial growth factor (anti-VEGF) agents, which are used alongside treatments such as steroids, laser photocoagulation, and vitrectomy, have improved the treatment of vision-threatening DR [3]. However, anti-VEGF treatments require frequent intravitreal injections, and over half of patients with DME do not respond or respond inadequately [4]. Thus, the need for novel, effective, non-invasive treatments for the sight-threatening manifestations of DR remains, especially as the incidence of diabetes rises around the world [5], even if some of these treatments have potential issues of their own such as poor patient adherence and systemic side effects.

While the mechanisms underlying the pathogenesis of DR are complex and not fully understood, evidence suggests that hypoxia plays an important role in its development and progression [6–8]. Diabetes is associated with deficits in oxygen delivery to the retina [8, 9], leading to retinal hypoxia that may be evident even before development of clinically detectable microvascular damage [10–12]. The microvasculopathy associated with diabetes leads to further retinal ischemia and tissue hypoxia, which results in upregulation of cytokines, particularly VEGF [13]. Increases in these cytokines lead to both vascular leakage, in the form of DME, and retinal neovascularization, which is the hallmark of PDR [7]. Anti-VEGF therapies work by blocking VEGF, a cytokine that plays a key role in hypoxia-mediated neovascularization and vascular leakage [14]. A role for hypoxia in DR is further supported by a study that found that 3 months of nasal oxygen therapy reduced macular thickness in DME patients [15]. Collectively, this evidence suggests that reducing retinal hypoxia may help ameliorate DR.

During dark adaptation (i.e., during sleeping), the photoreceptors are most metabolically active, as they consume large amounts of oxygen and adenosine triphosphate (ATP) to maintain the electrochemical gradients associated with the “dark current” [16]. During light adaptation, oxygen consumption is decreased by 40 to 60% relative to the dark adapted state [17]. It has been postulated that preventing complete dark adaptation may decrease the hypoxia associated with DR and be effective in its treatment [18]. Based on this rationale and positive earlier clinical studies [18, 19], a recent phase 3 trial investigated whether wearing a light mask to prevent dark adaptation while sleeping would reduce retinal thickness in patients with DME [20]. Although this study did not find the light mask to be effective, other methods of decreasing retinal hypoxia by preventing dark adaptation are being investigated.

Recent research indicates that emixustat HCl (emixustat) reduces the demand for oxygen in the retina, decreasing hypoxia, and may thus have a role in the treatment of DR. Emixustat is an orally available small molecule that inhibits a visual cycle enzyme, retinal pigment epithelium–specific 65-kDa protein (RPE65), resulting in slowing of the visual cycle [21]. This leads to a reduction in the availability of 11-*cis*-retinal to bind with opsin and therefore increased levels of free opsin (apo-opsin). Apo-opsin can produce low-level phototransduction, preventing complete dark adaptation and reducing the oxygen- and energy-intensive dark current [22–25]. A recent work in rats has shown that emixustat reduces levels of retinal cation influx and oxygen consumption in the dark, indicating that it reduces the metabolically demanding dark current [26]. In addition, emixustat produces a dose-dependent decrease in neovascularization in a retinopathy of prematurity rodent model which is driven by retinal hypoxia [27]. Thus, emixustat may be a promising candidate for the treatment of DR.

In this phase 2 trial, the primary objective was to evaluate the effects of 84 days of treatment with oral emixustat on pro-angiogenic and inflammatory cytokines in the aqueous humor of subjects with PDR, with or without DME. Past research has shown that levels of pro-angiogenic and inflammatory cytokines, including interleukin (IL)-1β, IL-6, IL-8, interferon-gamma-inducible protein (IP)-10, monocyte chemo-attractant protein (MCP)-1, transforming growth factor (TGF)β-1, and VEGF, are elevated in the intraocular fluid of subjects with PDR [28–34]. In addition, pro-angiogenic factors such as VEGF have been shown to be hypoxia-responsive [35]. If emixustat reduces oxygen demand in the retina, treatment with emixustat may reduce levels of aqueous humor pro-angiogenic and inflammatory cytokines associated with PDR. The study’s secondary objective was to evaluate the effects of emixustat on ophthalmic assessments affected in DR.

## Methods

### Study design

This multicenter, randomized, double-masked, placebo-controlled trial was conducted from April 2016 to November 2017 at 8 study sites in the USA, in accordance with the Declaration of Helsinki and with Health Insurance Portability and Accountability Act regulations. Institutional Review Board (IRB)/Ethics Committee approval was obtained, and the trial protocol and informed consent form were approved by Alpha IRB (San Clemente, CA). All subjects provided written informed consent before study-specific procedures began.

### Participants

Subjects who were able to provide informed consent were eligible for the study if they were ≥ 18 and ≤ 85 years of age; had a documented diagnosis of type 1 or type 2 diabetes mellitus; had PDR in their study eye, with or without DME, for which, in the investigator’s judgment, interventional treatment could be safely deferred for at least 4 weeks after the day 1 visit; and had a best-corrected visual acuity (BCVA) letter score of ≥ 24 (approximate Snellen equivalent 20/320) in their study eye as determined using the Early Treatment Diabetic Retinopathy Study (ETDRS) method. Additional entry criteria included no prior pan-retinal photocoagulation in the study eye; no intravitreal injection of an anti-VEGF agent in the study eye during the 3 months prior to randomization; and no intravitreal or peri-bulbar injection of a corticosteroid in the study eye during the 4 months prior to randomization. Subjects were excluded from the study if they had poor glycemic control; this included patients who initiated intensive insulin treatment in the 4 months prior to screening or planned to do so during the 4 months of the study. Additional exclusions included implantation in the study eye of fluocinolone acetonide (Iluvien^®^) in the 30 months prior to randomization, or dexamethasone (Ozurdex^®^) in the 4 months prior to randomization. Subjects with macular edema in the study eye due to anything other than diabetes were also excluded. If both eyes of a subject qualified for the study, the investigator selected the eye that, in his or her opinion, had the least potential to require pan-retinal photocoagulation, anti-VEGF therapy, or local corticosteroids during the study.

### Randomization and masking

Subjects were randomly assigned to the emixustat or placebo arm in a 1:1 ratio. The randomization schedule was computer-generated using permuted blocks and included stratification by DME status in the study eye (with or without). It was uploaded to an interactive web response system where qualified subjects were sequentially assigned to the next available randomization number by a member of the study site staff. The appropriate masked medication was then given to the subject using a preassigned kit number. Subjects, investigators, staff, and sponsor personnel involved in the conduct and monitoring of the study were masked to the identity of treatment until after the final database was locked. If a screened subject was not randomized and dosed by day 30 after screening, the subject could be re-screened by repeating all of the screening procedures.

### Procedures

**Fig. 1:**
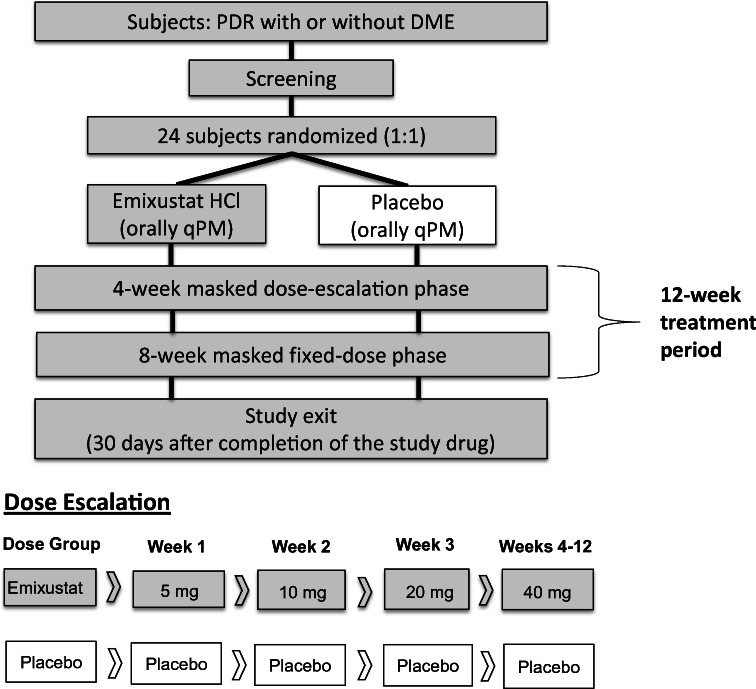
Study design, including diagram of dose escalation phase. Mock dose escalation was performed in the placebo arm to maintain masking. Subjects who did not tolerate dose escalations could return to the last tolerated dose, but a dose reduction could be undertaken only once and only during weeks 2, 3, or 4. After week 4, all subjects were held at a stable dose. DME, diabetic macular edema; PDR, proliferative diabetic retinopathy; qPM, taken every evening

Subjects were treated once daily with orally administered emixustat or placebo in the evening, for a total of 84 days (Fig. 1). Subjects assigned to the emixustat arm received 5 mg emixustat for the first week; then, the dose was doubled on a weekly basis to 10 mg (week 2), 20 mg (week 3), and 40 mg (weeks 4 through 12). Subjects who did not tolerate dose escalations due to adverse events (AEs) were returned to and held stable at the last tolerated dose. After week 4, all subjects were held at a stable dose for the remainder of the study. Dose reduction could only be undertaken once; was only available at the week 2, 3, or 4 visits; and was only taken if the subject experienced an AE that would otherwise lead to discontinuation of the study drug, in the opinion of the investigator. All subjects received 4 identical appearing tablets every day. For example, in week 1, subjects in the emixustat arm received two placebos and two 2.5-mg tablets; and in week 2, two placebos and two 5.0-mg tablets. Doses were within the range previously assessed for safety in a phase 1b clinical trial [36]. Subjects in the placebo arm also received 4 tablets daily and were mock-titrated on the same schedule as those in the emixustat arm. Placebo tablets were packaged identically, in tamper-proof blister packaging, to maintain masking.

Scheduled visits included screening (within 30 days prior to baseline); baseline (day 1); days 8, 15, 22, and 29; and days 57 and 85. The day 85 visit occurred 1 day after the last dose of study drug. The study exit visit was performed 30 days after the last dose of study drug. At each visit, subjects were queried regarding compliance with their treatment regimen, and their remaining pills were counted. Unscheduled visits could be performed as necessary due to AEs or for other reasons.

Efficacy assessments included aqueous humor sampling at day 1 and day 85 for the levels in the study eye of 7 aqueous humor cytokines (IL-1β, IL-6, IL-8, IP-10, MCP-1, TGFβ-1, and VEGF); color fundus photographs (CFP) (days 1 and 85); spectral domain optical coherence tomography (SD-OCT) (days 1, 29, 57, and 85); and BCVA (days 1, 29, 57, and 85). Aqueous humor samples (50 μL) were obtained by paracentesis and shipped frozen on dry ice the same day to PharmOptima Laboratories (Portage, MI, USA), where cytokines were evaluated by multiplex immunoassay on the Meso Scale Discovery platform. The SD-OCT machine used at all sites was the Heidelberg SPECTRALIS^®^, Heidelberg Engineering GmbH (Heidelberg, Germany).

Additional safety assessments included AE assessments, physical exams, electrocardiography, vital signs, pregnancy tests, clinical laboratory tests (chemistry, hematology, coagulation, urinalysis, hemoglobin A1c), low-luminance BCVA (2.0 log neutral density filter placed before the eye) [37], slit-lamp biomicroscopy, intraocular pressure, gonioscopy, and dilated ophthalmoscopy.

### Efficacy outcomes

The preplanned primary study endpoints were mean changes from baseline (day 1) to day 85 in the level of the 7 aqueous humor cytokines. Analyses were planned for both the absolute changes and the percent changes from baseline. However, only 5 cytokines were analyzed because tests for IP-10 and MCP-1 failed accuracy and stability testing during assay development and were therefore dropped from the study. Preplanned secondary efficacy endpoints included the mean changes from baseline to day 85 in BCVA and, as determined by a central image reading center (Ocular Imaging Research and Reading Center, Omaha, NE, USA), the area of retinal neovascularization (assessed using CFP), and central subfield thickness (CST) in subjects with DME at baseline (assessed using SD-OCT). Additional efficacy endpoints investigated in ancillary analyses included mean changes from baseline to day 85 in CST in all subjects, and total macular volume (TMV) in all subjects and in the subset of subjects with DME at baseline.

### Safety outcomes

All subjects who received at least 1 dose of the study drug were included in the safety analysis. Assessment of safety was based on the summaries of ocular and non-ocular AEs, BCVA, low-luminance BCVA, ophthalmic examination findings (slit-lamp biomicroscopy, dilated ophthalmoscopy, intraocular pressure, and gonioscopy), SD-OCT, vital signs, physical examination findings, electrocardiograms (ECGs), and clinical laboratory values. For all AEs, information was gathered on severity, onset and resolution dates and times, frequency, seriousness, relationship to study drug, action taken, outcome, location, and whether the AE caused the subject to discontinue the study. All AEs had their verbatim terms mapped to the corresponding thesaurus terms using the Medical Dictionary for Regulatory Activities (MedDRA^®^) coding dictionary, Version 19.0. Subject participation was discontinued if investigators determined that rescue treatment, in the form of pan-retinal photocoagulation, anti-VEGF therapy, or local corticosteroids, was necessary in the study eye. All subjects who discontinued treatment prematurely were withdrawn from the study, and subjects could withdraw for any reason or at any time.

### Statistical methods

Power calculations indicated that 8 subjects per arm would yield 80% power to detect a difference between the emixustat and placebo arms of the study for each aqueous humor cytokine, using a 2-sample *t* test and a 2-sided significance level of 0.10, and assuming the magnitude of the standard deviation is approximately 75% of the treatment difference. To account for early terminations, 2 additional subjects were planned to be enrolled per treatment arm, for a total of 10 subjects per arm.

A modified intent-to-treat population was analyzed to assess efficacy. This population included all randomized subjects. All subjects receiving emixustat were combined into one group. For subjects missing day 85 (week 12) efficacy values, the last observations (i.e., the measurements collected at the early termination visit) were carried forward and used to impute the missing values, but only for subjects who had been on the study drug for at least 2 months (60 days). If a subject did not have a day 85 value and the value could not be imputed, that subject was not included in the analysis for that particular variable. Primary, secondary, and ancillary endpoints were summarized using standard quantitative summary statistics (sample size, mean, SD, median, minimum, and maximum) and qualitative summary statistics (frequency counts and percentages). Endpoints were assessed using 2-sided, 2-sample *t* tests and 2-sided 90% *t*-distribution confidence intervals around the difference in means between treatment groups. To assess changes from baseline in aqueous humor levels of the 5 evaluable cytokines, both absolute and percent changes were analyzed. Additionally, for quantitative measures, 1-sample *t* tests were performed on the change from baseline values within a treatment group. Due to the exploratory nature of this study, no adjustments for multiple testing were conducted. All hypotheses were tested using a 2-sided significance level of 0.10.

## Results

**Fig. 2:**
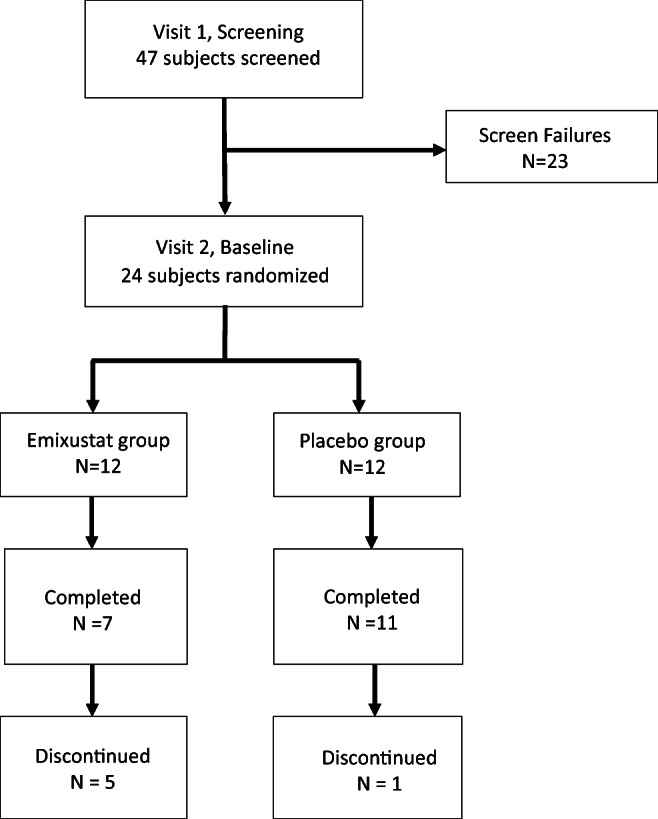
Disposition of study subjects

The investigators screened 47 subjects (Fig. 2). Of these, 23 subjects failed screening, 18 because of prohibited medications or laboratory test findings that did not meet the entry criteria. The 24 remaining subjects were randomized, 12 to the emixustat arm and 12 to the placebo arm. Of the subjects in the emixustat group, 2 were titrated to and remained at the maximum dose of 40 mg, until both discontinued the study after approximately 60 days of dosing. Seven subjects in the emixustat group completed the study, including all 3 who took 20 mg of the drug during the fixed-dose phase of the study, 3 of the 5 who took 10 mg, and 1 of the 2 who took 5 mg. In the emixustat group, 3 subjects withdrew from the study because of AEs and 2 subjects withdrew consent. One subject in the placebo group withdrew consent prior to dosing; all participants in the placebo group who initiated dosing (*n* = 11) completed the study. No subject in either group required rescue treatment. Changes in cytokine concentrations, the primary outcome, and changes in ophthalmic assessments were analyzed in 9 subjects in the emixustat group and 11 in the placebo group.

Demographic and baseline characteristics were well balanced between the treatment arms (Table 1). Subjects’ mean age was 49.8 (SD 9.5) years, and 50% of subjects (12/24) were women. With regard to race and ethnicity, 66.7% of subjects (16/24) were Hispanic, 12.5% (3/24) were black, and 20.8% (5/24) were non-Hispanic whites. The mean hemoglobin A1c level of subjects was 8.98% (SD 1.66%). Mean treatment compliance values over the course of the study, as assessed by the percentage of tablets taken relative to the total number of tablets expected to be taken, were different between the groups: 84.8% (SD 23.2%) for the emixustat group versus 97.7% (SD 3.6%) for the placebo group. Nine of 12 subjects (75%) in the emixustat group and 11 of 11 subjects (100%) in the placebo group had > 80% compliance.

**Table 1:** Demographic and baseline data for all randomized subjects

Characteristic	Emixustat (*N* = 12)	Placebo (*N* = 12)	All subjects (*N* = 24)
Age			
Mean (SD)	52.1 (7.1)	47.4 (11.3)	49.8 (9.5)
Median	53.0	49.5	51.5
Min, max	37, 60	28, 60	28, 60
Sex, *n* (%)			
Male	5 (41.7)	7 (58.3)	12 (50.0)
Female	7 (58.3)	5 (41.7)	12 (50.0)
Ethnicity, *n* (%)			
Hispanic	10 (83.3)	6 (50.0)	16 (66.7)
Non-Hispanic	2 (16.7)	6 (50.0)	8 (33.3)
Race, *n* (%)			
White	11 (91.7)	10 (83.3)	21 (87.5)
Black or African American	1 (8.3)	2 (16.7)	3 (12.5)
Hemoglobin A1c (%)			
Mean (SD)	8.98 (1.64)	8.98 (1.76)	8.98 (1.66)
Median	9.25	9.85	9.50
Min, max	6.0, 11.1	5.8, 11.0	5.8, 11.1

With regard to the study’s primary outcome, levels of pro-angiogenic and inflammatory cytokines in the aqueous humor, no statistically significant changes from baseline were detected between the 2 groups (Table 2). For IL-1β, all values were below the lower limit of detection (0.95 pg/mL) and were recorded as zero. For the remaining 4 cytokines (IL-6, IL-8, TGFβ-1, and VEGF), no statistically significant differences between treatment groups were detected with regard to either absolute or percent changes from baseline. However, for VEGF, the median change from baseline (− 70.0 pg/mL for emixustat versus + 42.7 pg/mL for placebo) and median percentage change from baseline (− 11.8% for emixustat versus + 6.7% for placebo) demonstrated numerical reductions in the emixustat but not in the placebo group.

**Table 2:** Mean and median changes in cytokine concentrations in the emixustat and placebo groups, in the study eye

Cytokine concentration (pg/mL)	Emixustat group (*N* = 9)^a^				Placebo group (*N* = 11)^b^				*P* value
	Mean baseline value (SD)	Mean day 85 value (SD)	Mean change (SD)	Median change (Q1, Q3)	Mean baseline value (SD)	Mean day 85 value (SD)	Mean change (SD)	Median change (Q1, Q3)	Difference in mean changes between groups
IL-1β^c^	0.0 (0.0)	0.0 (0.0)	0.0 (0.0)	0.0 (0.0)	0.0 (0.0)	0.0 (0.0)	0.0 (0.0)	0.0 (0.0)	–
IL-6	7.3 (10.1)	52.9 (141.3)	+ 45.6 (137.1)	+ 0.6 (0.0, + 10.0)	6.5 (4.7)	11.4 (12.0)	+ 4.9 (12.6)	+ 4.2 (− 5.1, + 9.6)	0.34
IL-8	11.3 (4.5)	15.5 (5.4)	+ 4.2 (5.1)	+ 2.8 (+ 1.4, + 4.9)	17.9 (19.8)	20.4 (22.9)	+ 2.6 (4.6)	+ 2.8 (− 0.9, + 4.7)	0.46
TGFβ-1	69.3 (25.3)	49.4 (31.3)	− 19.9 (32.4)	− 15.4 (− 30.8, 0.0)	57.7 (32.8)	40.2 (35.7)	− 17.5 (43.7)	− 22.6 (− 58.2, + 10.7)	0.89
VEGF	509.6 (202.1)	471.5 (181.0)	− 38.0 (115.1)	− 70.0 (− 84.9, + 23.9)	714.4 (528.9)	689.6 (339.8)	− 24.8 (277.2)	+ 42.7 (− 102.9, + 74.6)	0.90

^a^The sample size was 9 in the emixustat group because 1 subject did not have a post-baseline aqueous humor sample taken, and 2 subjects discontinued the study early and were on the study drug for less than 60 days

^b^The sample size was 11 in the placebo group because 1 subject did not have a post-baseline aqueous humor sample taken

^c^All values were below the lower limit of detection and are recorded as zero

For prespecified secondary endpoints—including the area of retinal neovascularization, CST in subjects with DME, and BCVA—no statistically significant changes from baseline were detected within or between the 2 groups (Table 3). However, mean CST in subjects with DME did improve in the emixustat but not in the placebo group (− 21.2 versus + 46.0 μm, respectively; *P* = 0.15 for the difference between the groups).

**Table 3:** Mean and median changes in ophthalmologic assessments in the emixustat and placebo groups, in the study eye

Measurement	Mean baseline value (SD)	Mean day 85 value (SD)	Mean change (SD)	Median change (Q1, Q3)	Mean baseline value (SD)	Mean day 85 value (SD)	Mean change (SD)	Median change (Q1, Q3)	*P* value Difference in mean changes between groups
All subjects	Emixustat group (*N* = 9)				Placebo group (*N* = 11)				
CST (μm)	334.2 (134.1)	322.3 (140.2)	− 11.9 (36.5)	− 6.0 (− 15, + 2)	297.2 (46.3)	333.4 (103.9)	+ 36.2 (69.0)	+ 9.0 (+ 1, + 38)	0.076*
TMV (mm^3^)	9.90 (1.71)	9.78 (1.71)	− 0.13 (0.19)	− 0.08 (− 0.20, − 0.01)	9.39 (1.27)	9.63 (1.56)	+ 0.23 (0.41)	+ 0.07 (− 0.03, + 0.46)	0.026*
Area of retinal neovascularization (mm^2^)^a^	5.52 (7.89)	5.98 (9.91)	+ 0.46 (3.11)	+ 0.70 (− 1.55, + 1.79)	1.91 (2.29)	3.07 (3.82)	+ 1.16 (2.10)	+ 0.06 (0.00, + 1.86)	0.60
BCVA (letters)	75.2 (6.0)	74.1 (8.2)	− 1.1 (8.9)	+ 2.0 (− 5, + 4)	79.2 (10.4)	78.8 (10.1)	− 0.4 (4.1)	0 (− 2, + 3)	0.80
Subjects with DME	Emixustat group (*N* = 5)				Placebo group (*N* = 7)				
CST (μm)	388.6 (164.6)	367.4 (180.5)	− 21.2 (48.2)	− 12.0 (− 22, − 6)	306.3 (51.2)	352.3 (126.8)	+ 46.0 (86.6)	+ 8.0 (+ 1, + 79)	0.15
TMV (mm^3^)	10.65 (1.97)	10.42 (2.03)	− 0.23 (0.19)	− 0.20 (− 0.29, − 0.08)	9.84 (1.31)	10.09 (1.68)	+ 0.26 (0.50)	+ 0.07 (− 0.16, + 0.46)	0.07*

*CST*, central subfield thickness; *BCVA*, best-corrected visual acuity; *DME*, diabetic macular edema; *TMV*, total macular volume

^a^For area of retinal neovascularization, sample sizes were 6 in the emixustat group and 10 in the placebo group, as the reading center could not determine this measurement at baseline and day 85 in all patients

*Statistically significant difference at the predefined level of 0.10 for this pilot study

A post hoc analysis conducted among all subjects (with or without DME) showed statistically significant differences between groups in the mean changes from baseline in CST (− 11.9 μm for emixustat versus + 36.2 μm for placebo; *P* = 0.076) and TMV (− 0.13 mm^3^ for emixustat versus + 0.23 mm^3^ for placebo; *P* = 0.026), both favoring emixustat (Table 3).

Of the 23 subjects in the safety population (12 in the emixustat group, 11 in the placebo group), 21 reported at least one AE. A total of 139 AEs were reported, with 79 in the emixustat group and 60 in the placebo group. All subjects in the emixustat group experienced at least 1 ocular AE, whereas 9 of 11 (81.8%) subjects in the placebo group did. In addition, 6 of 12 subjects in the emixustat group and 4 of 11 subjects in the placebo group experienced at least 1 non-ocular AE. Delayed dark adaptation, visual impairment, visual acuity reduced, chromatopsia, and erythropsia occurred more frequently in the emixustat group (Table 4). Blurred vision was more common in the placebo group.

**Table 4:** Adverse events with incidence ≥ 15% for all study subjects

Adverse event	Emixustat (*N* = 12), *n* (%)	Placebo (*N* = 11), *n* (%)	All subjects, (*N* = 23), *n* (%)
Any adverse event	12 (100)	9 (81.8)	21 (91.3)
Delayed dark adaptation	9 (75.0)	1 (9.1)	10 (43.5)
Visual impairment	6 (50.0)	1 (9.1)	7 (30.4)
Vision blurred	1 (8.3)	6 (54.5)	7 (30.4)
Blindness day	4 (33.3)	3 (27.3)	7 (30.4)
Vitreous floaters	4 (33.3)	2 (18.2)	6 (26.1)
Visual acuity reduced	4 (33.3)	1 (9.1)	5 (21.7)
Chromatopsia	4 (33.3)	0	4 (17.4)
Visual acuity tests abnormal^a^	2 (16.7)	2 (18.2)	4 (17.4)
Vitreous hemorrhage	3 (25.0)	1 (9.1)	4 (17.4)

^a^For all 4 subjects, “visual acuity tests abnormal” refers to decreases in low-luminance best-corrected visual acuity

Two subjects in the emixustat group and 2 subjects in the placebo group experienced 1 or more grade 3 (severe) AEs. In the emixustat group, these included delayed dark adaptation, retinal detachment, retinal hemorrhage, and visual acuity reduced. Only delayed dark adaptation was considered by the investigator to be related to the study drug. In the placebo group, they included photophobia, macular fibrosis, and headache. One subject in the emixustat group experienced a grade 4 (life-threatening) AE, which was determined by the investigator not to be related to the study drug. The AE was vitreous hemorrhage; the severity of this AE is likely to have been incorrectly entered by the investigator. Two subjects, both in the placebo group, experienced serious AEs, but neither withdrew from the study. Both were non-ocular in nature (moderate cellulitis and severe hyperkalemia) and were not considered related to the study drug. In 3 cases, all in the emixustat group, an ocular AE led to study withdrawal (delayed dark adaptation [2 subjects] and visual impairment).

More subjects in the emixustat group experienced ≥ 15 letter decreases in NL-BCVA in 1 or both eyes at 1 or more visits than those in the placebo group. The majority of NL-BCVA decreases in both treatment groups resolved by study end and were coincident with worsening vitreous hemorrhage and/or significant increases in CST. Low-luminance BCVA decreases of ≥ 10 letters occurred more frequently in the emixustat group in both the study eye (50% (6/12) versus 18% (2/11)) and the non-study eye (50% (6/12) versus 0% (0/11)). Finally, hemoglobin A1c levels marginally decreased over the course of the study, falling 4.2% in the emixustat group and 3.8% in the placebo group.

## Discussion

The primary objective of this pilot study was to evaluate the effects of oral emixustat on pro-angiogenic and inflammatory cytokines in the aqueous humor of subjects with PDR, over an 84-day treatment period. No statistically significant differences between treatment groups were observed for changes from baseline in any of the cytokines tested (IL-6, IL-8, TGFβ-1, VEGF). However, in the emixustat group, median VEGF levels decreased from baseline, whereas they increased in the placebo group. An emixustat-linked positive trend was also observed for a secondary outcome, the change in CST in subjects with DME, though statistical significance was not reached, likely due to the small number of subjects. Finally, in a post hoc analysis conducted among subjects with and without DME at baseline, a statistically significant difference between the treatment groups in the change from baseline to day 85 was observed for both CST and TMV, with improved values in the emixustat but not in the placebo group.

Cytokine values in this study were consistent with those from previous studies in similar patient populations. All baseline values for each aqueous humor cytokine fell within the ranges previously reported for diabetic patients [29, 33]. In addition, previous studies have reported a large amount of between-subjects variability in the levels of these cytokines [29, 30, 33], similar to what was observed in this trial. The clinical relevance of the reduction in VEGF levels (11.8%) in response to emixustat treatment is unclear; for comparison, a 97.4% reduction in median aqueous humor VEGF levels was seen over 2 months in DME patients treated with the VEGF inhibitor ranibizumab [38].

That statistically significant changes favoring emixustat for both CST and TMV were detected indicates that emixustat treatment is associated with beneficial changes in this patient population, though the clinical relevance of these changes is unclear. The decrease in VEGF levels after emixustat treatment, though small, may play a role in these decreases in retinal thickness. However, the failure to detect statistically significant changes in the tested cytokines in the presence of improved CST and TMV values suggests that emixustat has a beneficial effect on retinal thickness through some mechanism independent of these cytokines. For example, emixustat may decrease vascular permeability by reducing all-*trans*-retinal-mediated toxicity, as found in experiments in a retinal ischemia-reperfusion mouse model [39].

The safety profile of emixustat in the PDR population is similar to that demonstrated in prior studies of emixustat in healthy volunteers and patients with geographic atrophy (GA) secondary to age-related macular degeneration. In these studies, the most common AEs (delayed dark adaptation and visual impairment) were ocular in nature and most likely reflected the drug’s mechanism of action (RPE65 inhibition) [36, 40, 41]. Decreases in low-luminance BCVA, a measure of mesopic cone function, seen after emixustat treatment both in the current study and in studies of patients with GA, may be due to an indirect effect on cones through rod-cone interactions [41]. In the current study, the drop-out rate among emixustat subjects was high (5/12, 41.7%) even with the allowance for dose reductions, and drop-outs were primarily due to emixustat-related AEs, as has been seen in prior studies [40, 41].

This study has important limitations. First, a large proportion of subjects in the emixustat group (5 of 12) did not complete the study. In addition, because of the small sample size and exploratory nature of this study, control for multiple testing was not implemented when performing statistical analyses. As a result, the preliminary findings regarding emixustat-associated improvements in mean and median VEGF levels, CST, and TMV should be regarded with caution until they can be replicated in a larger study.

In conclusion, although this pilot study did not demonstrate statistically significant differences between the emixustat and placebo groups for any of the pro-angiogenic and inflammatory cytokines investigated, a decrease in aqueous humor VEGF levels was observed in the emixustat but not in the placebo group. Moreover, among the prespecified secondary endpoints, an improvement in CST among subjects with DME was observed, which was supported by statistically significant improvements observed in a post hoc analysis of CST and TMV among all subjects. These findings warrant further investigation of emixustat treatment for DR.

## Data Availability

The data generated during the current study are not available at this time since the drug being tested is investigational, and the data are proprietary and may be part of future regulatory submissions.
